# The effect of enhanced recovery protocols on postoperative rehabilitation in orthopedic surgery patients

**DOI:** 10.1097/MD.0000000000041143

**Published:** 2025-01-17

**Authors:** Hui Cao, Zhaoxu Huang, Hua Yuan, Xuehui Hu, Zhanli Fu, Xiaohuan Song, Meixia Zhang, Xia Du, Zhuoyu Long

**Affiliations:** a Rehabilitation Medicine Department, The First Affiliated Hospital of Air Force Medical University, Xi’an, China; b Department of Anesthesiology, The First Affiliated Hospital of Air Force Medical University, Xi’an, China; c Nursing Department, The First Affiliated Hospital of Air Force Medical University, Xi’an, China; d The Second Department of Orthopaedics, NO.3 Hospital of Xi’an City, Xi’an, China; e Anesthesia and Surgery Department, Xi’an Fengcheng Hospital, Xi’an, China.

**Keywords:** enhanced recovery, fracture healing, joint function, orthopedics, rehabilitation process

## Abstract

Orthopedics is a critical hospital department that has experienced shifts in the spectrum of orthopedic conditions due to societal advancements in recent years. While surgical interventions are effective in restoring fracture function, perioperative care remains a key factor in optimizing patient recovery. This study aims to assess the impact of orthopedic rehabilitation care based on the enhanced recovery after surgery (ERAS) protocol on postoperative rehabilitation. A total of 836 patients who underwent orthopedic surgery from June 2023 to June 2024 were included in the study. They were divided into a control group, which received standard care, and an observation group, which received care interventions based on ERAS principles. Recovery outcomes were assessed by measuring hospital stay duration, fracture healing time, joint function recovery time, and the incidence of complications in both groups. The observation group showed significantly shorter hospital stays, faster fracture healing times, and quicker joint function recovery compared with the control group. Furthermore, the incidence of complications was notably lower in the observation group. Postoperative joint function recovery scores and patient satisfaction were also higher in the observation group, with statistically significant differences between the 2 groups (*P* < .05). ERAS-based care enhances orthopedic recovery by reducing hospital stays, accelerating functional recovery, lowering complications, and improving patient satisfaction, proving its effectiveness in perioperative cares, and should be widely implemented in orthopedic rehabilitation.

## 1. Introduction

Orthopedics plays a vital role in comprehensive hospitals, focusing on the study of the musculoskeletal system’s structure, function, and related disorders. It also explores the efficacy of medications, surgical techniques, and physical therapies in preserving and restoring normal musculoskeletal health.^[1,2]^ Diseases such as bone and joint tuberculosis, osteomyelitis, and poliomyelitis have significantly declined, while traumatic fractures, often linked to traffic accidents and other incidents, have markedly increased.^[3]^ In clinical practice, customized surgical strategies for fractures have proven to enhance treatment outcomes effectively, aiding in fracture reduction and functional recovery. However, the trauma of surgery still impacts the patient’s physiology. Without coordinated and effective perioperative care, orthopedic surgery patients often face complications such as infection, thrombosis, and others, which can diminish the effectiveness of the surgery and hinder postoperative recovery.^[4]^

Currently, accelerated recovery after surgery care is widely used in orthopedics. It was first proposed by Danish surgeon Kehlet.^[5]^ The accelerated recovery after surgery-based care model refers to the use of comprehensive clinical medical knowledge by nurses during surgery to adjust and intervene in the patient’s physiological and psychological aspects, aiming to reduce surgical stimulation, prevent related complications, and promote postoperative recovery.^[6]^ We hypothesize that the concept of accelerated recovery after surgery can improve postoperative orthopedic recovery. This study aims to explore the impact of orthopedic rehabilitation care based on accelerated recovery after surgery concepts on the functional recovery and rehabilitation process of postoperative patients.

## 2. Materials and methods

### 2.1. General information

This study was approved by the Ethics Committee of NO.3 Hospital of Xi’an City. A total of 836 patients who underwent orthopedic surgery at our hospital between June 2023 and June 2024 were selected for this study. The patients were divided into 2 groups according to their admission time: a control group and an observation group, with 418 cases in each group. In the control group, there were 320 females and 98 males, aged 48 to 90 years, with an average age of 74.25 ± 10.24 years. The causes of injury are given as follows: 390 cases of common fall-related fractures, 18 cases of fractures due to external force, and 10 cases of fractures caused by traffic accidents. In the observation group, there were 280 females and 138 males, aged 49 to 91 years, with an average age of 74.51 ± 10.54 years. The causes of injury are given as follows: 380 cases of common fall-related fractures, 28 cases of fractures due to external force, and 8 cases of fractures caused by traffic accidents. There was no statistically significant difference in the general information between the 2 groups (*P* > .05), indicating comparability.

### 2.2. Inclusion and exclusion criteria

#### 2.2.1. Inclusion criteria

Patients who met the relevant diagnostic criteria in “New Advances in Clinical Orthopedic Diagnosis and Treatment”^[7]^ exhibited typical symptoms of sudden renal or ureteral colic accompanied by hematuria and difficulty in urination, had complete clinical data, were undergoing orthopedic surgery for the first time, had strong compliance, and were able to communicate well with medical staff. Both the patients and their families were informed about the study and signed informed consent forms.

#### 2.2.2. Exclusion criteria

Patients who withdrew from treatment midway, had coagulation disorders, or had significant organ dysfunction were excluded, as well as those unable to cooperate with medical staff to complete nursing care.

### 2.3. Nursing methods

In this study, patients in the control group received clinical routine care interventions, including vital signs monitoring and basic nursing care. Patients in the observation group received enhanced recovery care interventions, which included the following.

#### 2.3.1. Psychological care intervention

Postoperative immobility in the affected limb can easily lead to irritability and anxiety in patients. Nurses should provide timely psychological counseling to eliminate negative emotions and introduce successful cases to boost the patient’s confidence in the treatment and encourage cooperation.

#### 2.3.2. Rehabilitation training intervention

Patients were guided to perform early bed rehabilitation exercises by contracting muscles and bending joints. Later stages involved joint function recovery through weight-bearing exercises and flexibility training. Regular massage of the affected joints and maintaining a clean and tidy bed environment were also emphasized to prevent bedsores.

#### 2.3.3. Pain management intervention

For patients with mild postoperative pain, pain relief was managed through methods such as distraction and local cold compresses. For those with more severe pain, pain relief was managed through medication and physical therapy.

#### 2.3.4. Dietary intervention

Patients were restricted from water and food for the first 6 hours post-operatively. After 24 hours, they were guided to consume semiliquid food, gradually transitioning to a normal diet. The diet was mainly high in protein and nutrients to enhance the patient’s resistance to disease.

### 2.4. Observation indicators

The postoperative recovery process of the 2 groups was observed. The functional recovery of the affected limb in both groups was assessed using the limb function (hip joint, wrist joint, and ankle joint) scoring standard, with a score out of 100 points. A higher score indicated better functional recovery. The degree of pain in both groups was compared. The visual analog scale (VAS) was used to assess pain before and after nursing care, with a total score of 0 to 10 points, where a higher score indicated more severe pain.^[8]^ Postoperative outcomes, including fracture healing time, joint recovery time, hospital stay, and nursing satisfaction, were compared between the 2 groups. Nursing satisfaction was assessed using a satisfaction survey, which included items on nursing effectiveness, quality of care, and service attitude, with a total score of 100 points.^[9,10]^ The incidence of complications, including pressure ulcers, venous thrombosis, tissue adhesion, and joint contracture, was compared between the 2 groups. The total complication rate = total number of complications/total cases × 100%.

### 2.5. Statistical analysis

Statistical analysis was conducted using SPSS software, with a 2-sided *P* value of <.05 considered statistically significant. Specific statistical methods included the following.

#### 2.5.1. Descriptive statistics

Descriptive analysis of general information in the observation and control groups was performed, with data presented as mean ± standard deviation (x ± s).

#### 2.5.1. Independent sample *t* test

This was used to compare continuous variables, such as hospital stay, fracture healing time, and joint recovery time, between the observation and control groups. The 95% confidence intervals for key variables were calculated to illustrate the precision of the data.

#### 2.5.2. Paired *t* test

It is applied to compare changes in the VAS scores before and after nursing care to assess the effects of nursing interventions.

#### 2.5.3. χ² test

This was used to compare categorical variables, such as complication rates and nursing satisfaction, between the observation and control groups.

#### 2.5.4. Adjustment for confounding factors

To control for potential confounding factors that might affect patient recovery outcomes (such as age, gender, type of injury, and severity), multivariable linear regression models were used for the primary outcome measures. Adjustments for these potential confounders allowed for a more accurate evaluation of the independent impact of enhanced recovery after surgery (ERAS) nursing interventions on postoperative recovery. All statistical results were reported with *P* values, and 95% confidence intervals were provided for key variables to enhance the scientific rigor and reliability of the conclusions.

**Figure 1.**
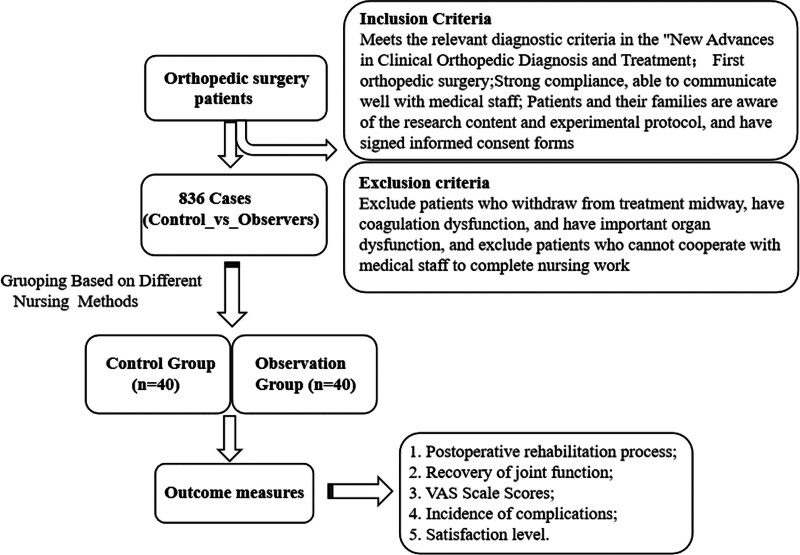
Flowchart of the study design. VAS = visual analog scale.

The study design overview is shown in Figure 1.

## 3. Results

### 3.1. Recovery status of patients in both groups

An evaluation of the recovery process for patients in both groups revealed that the observation group had significantly shorter hospital stays, fracture healing times, and joint function recovery times compared with the control group. The statistical analysis of these data showed that the differences were statistically significant (*P* < .05). Detailed information is provided in Table 1.

**Table 1:** Comparison of hospital stay, fracture healing time, and joint recovery time between the 2 groups of patients ($\bar{x}\pm s$).

Outcome measures	Observation group (n = 418)	Control group (n = 418)	t	*P*
Hospitalization time (95% CI), d	11.5 ± 4.1 (11.11–11.89)	20.9 ± 5.2 (20.4–21.4)	8.9778	.0000
Fracture healing time (95% CI), d	87.5 ± 32.6 (84.37–90.63)	112.7 ± 35.2 (109.32–116.08)	3.3219	.0014
Joint recovery time (95% CI), d	161.6 ± 41.7 (157.59–165.61)	259.7 ± 59.8 (253.95–265.45)	8.5104	.0000

CI = confidence interval.

### 3.2. Comparison of joint function recovery within 1 year post-surgery between the 2 groups

**Figure 2.**
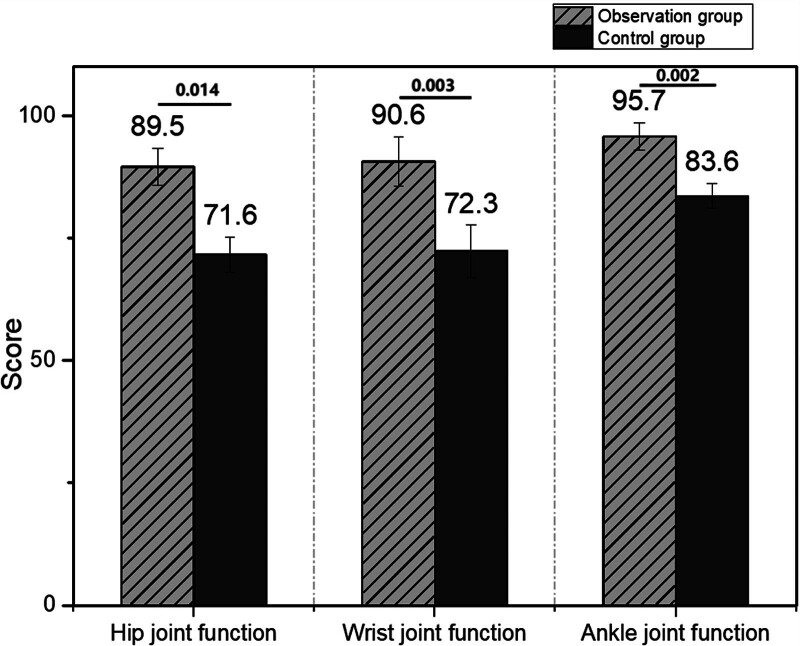
Comparison of joint function recovery scores 1 year post-surgery between the observation group and the control group.

The recovery of limb function in both groups was assessed. The control group showed significantly lower recovery scores across all joints (hip joint, 71.6 ± 3.6; wrist joint, 72.3 ± 5.3; and ankle joint, 83.6 ± 2.5) compared with the observation group (hip joint, 89.5 ± 3.8; wrist joint, 90.6 ± 5.1; and ankle joint, 95.7 ± 2.8). These differences were statistically significant (*P* < .05), as shown in Figure 2.

### 3.3. Comparison of VAS scores after rehabilitation nursing between the 2 groups

Before nursing care, the VAS score for the observation group was 4.76 ± 0.96, and for the control group, it was 4.81 ± 1.01. There was no statistically significant difference between the 2 groups (*P* > .05). After nursing care, the VAS score for the observation group was 1.70 ± 0.45, while, for the control group, it was 2.43 ± 0.61. Both groups had lower VAS scores compared with before nursing care, with the observation group having significantly lower scores than the control group. The difference was statistically significant (*P* < .05; Table 2).

**Table 2:** Comparison of VAS scores before and after nursing care between the 2 groups ($\bar{x}\pm s$).

Group	Before nursing	After nursing	t	*P*
Observation group (n = 418; 95% CI)	4.76 ± 0.96 (4.67–4.85)	1.70 ± 0.45 (1.66–1.74)	27.451	<.001
Control group (n = 418; 95% CI)	4.81 ± 1.01 (4.71–4.91)	2.43 ± 0.61 (2.37–2.49)	18.583	<.001
T	0.363	6.091		
*P*	.718	<.001		

CI = confidence interval, VAS = visual analog scale.

### 3.4. Comparison of complication rates 4 weeks post-surgery between the 2 groups

The total complication rate in the observation group was 22 (5.26%), which was significantly lower than the control group’s rate of 83 (19.81%). This difference was statistically significant (*P* < .05; Table 3).

**Table 3:** Postoperative complication rates (%) between the 2 groups.

Group	Pressure ulcer	Venous thrombosis	Tissue adhesion	Joint contracture	Always occurring
Observation group (n = 418)	0 (0.00)	11 (2.63)	11 (2.63)	0 (0.00)	22 (5.26)
Control group (n = 418)	21 (5.00)	21 (5.00)	31 (7.42)	10 (2.39)	83 (19.81)
χ^2^					4.114
*P*					.043

### 3.5. Nursing satisfaction post-surgery

The nursing satisfaction rates were 388 (92.82%) for the observation group and 321 (76.79%) for the control group. The comparison of nursing satisfaction between the 2 groups showed a statistically significant difference (χ² = 10.361; *P* = .002; Table 4).

**Table 4:** Postoperative nursing satisfaction rates (%) between the 2 groups.

Group	Very satisfied	Basically satisfied	Dissatisfied	Overall satisfaction rate
Observation group (n = 418)	221 (52.87)	167 (39.95)	30 (7.18)	388 (92.82)
Control group (n = 418)	129 (30.86)	192 (45.93)	97 (23.21)	321 (76.79)
χ^2^	10.719	1.805	10.361	10.361
*P*	.002	.231	.002	.002

## 4. Discussion

The concept of accelerated rehabilitation surgery (ERAS) has been widely used in the clinical nursing of surgical patients in recent years and has achieved remarkable nursing results. In orthopedic surgery, the need for perioperative care is higher because anesthesia, pain, and surgical stimulation increase intraoperative risks. ERAS can effectively reduce negative emotions and physiological stress caused by surgical trauma through a series of evidence-based nursing measures, such as preoperative psychological counseling, close cooperation during surgery, early postoperative rehabilitation guidance, and discharge education, and significantly reduce the incidence of postoperative complications such as infection and thrombosis.^[11–16]^ In this study, patients in the observation group had significantly shorter hospital stays, time spent in bed and time spent outside bed, lower treatment costs, better recovery of limb and joint function after surgery than the control group, and higher overall satisfaction of patients, indicating that ERAS nursing mode plays an important role in promoting postoperative rehabilitation and improving nursing quality of orthopedic surgery patients.

Previous studies have shown that participation in postoperative lower limb rehabilitation exercises can significantly accelerate postoperative recovery and improve patient satisfaction,^[17,18]^ which is consistent with our observation. Together, these findings support the results of our study and illustrate the importance of rehabilitation programs.

One of the core elements of the ERAS protocol is its focus on delivering comprehensive perioperative care that integrates psychological, rehabilitative, pain management, and dietary interventions tailored to the patient’s needs. In this study, psychological support played a pivotal role, as many patients undergoing orthopedic procedures experience high levels of anxiety, fear, and uncertainty about their recovery. Providing psychological counseling and reassurance helped alleviate these negative emotions, enabling patients to engage more fully with their care plans. This increased cooperation may be directly tied to the higher patient satisfaction scores observed in the ERAS group, as patients felt more supported and confident throughout their recovery journey.^[19,20]^ In addition, the structured rehabilitation interventions within the ERAS protocol, which included early bed-based exercises, gradual joint mobilization, and progressive strengthening activities, contributed significantly to improved functional recovery outcomes. By promoting regular joint movement early in the recovery phase, these interventions also prevented common postoperative complications such as joint contractures and pressure ulcers, which were notably lower in the ERAS group. These findings underscore the importance of integrating early, consistent rehabilitative exercises into postoperative care to optimize recovery and reduce adverse outcomes.^[21]^

Pain management was another critical factor. Patients in the ERAS group reported lower postoperative pain scores, likely due to the combined use of nonpharmacological and pharmacological pain relief strategies. This multifaceted approach not only reduced pain but also minimized the use of opioids, which are often associated with adverse effects in elderly orthopedic patients. The dietary guidelines within ERAS, focusing on high-protein, nutrient-dense foods, may also have enhanced the patients’ immune response and promoted healing, contributing to the faster recovery times observed.^[22]^

This study has several limitations that may affect the generalizability and interpretation of the findings. First, as a single-center study conducted at our institution, the results may not fully represent broader patient populations or varying clinical practices. This limitation may restrict the external validity and generalizability of our conclusions across other hospitals or regions. Second, patients were not randomly assigned to the observation or control groups; rather, group assignment was based on admission times. Although baseline characteristics were carefully compared and adjusted for potential confounding factors using multivariable linear regression, the lack of randomization introduces the possibility of selection bias, which may influence the observed differences between groups. Third, because this study involved a rehabilitation protocol requiring patient cooperation, strict blinding could not be implemented, potentially introducing performance or detection bias. This awareness might have impacted subjective outcomes, such as patient satisfaction and perceived pain levels. Future multicenter, randomized controlled trials with blinding are recommended to further validate these findings, reduce bias, and enhance the generalizability of the results.

## 5. Conclusion

The ERAS protocol significantly improves the postoperative outcomes of orthopedic surgery patients through comprehensive, evidence-based perioperative care. This study demonstrates that ERAS interventions, including psychological counseling, structured rehabilitation training, multimodal pain management, and tailored dietary support, effectively reduce hospital stays, accelerate fracture healing and joint function recovery, and lower complication rates.

These findings underscore the importance of adopting ERAS in orthopedic perioperative care to optimize patient recovery, improve care quality, and enhance overall satisfaction. Future research should explore broader applications of ERAS to further validate its efficacy across diverse surgical contexts.

## Author contributions

**Conceptualization:** Hui Cao, Zhaoxu Huang, Hua Yuan, Xuehui Hu, Xiaohuan Song, Zhuoyu Long

**Data curation:** Hui Cao, Zhaoxu Huang, Hua Yuan, Xuehui Hu, Xiaohuan Song, Meixia Zhang, Xia Du

**Formal analysis:** Hui Cao, Zhaoxu Huang, Hua Yuan, Xuehui Hu, Meixia Zhang, Xia Du

**Investigation:** Hui Cao, Zhaoxu Huang, Hua Yuan, Xuehui Hu, Zhanli Fu, Xiaohuan Song, Meixia Zhang, Xia Du, Zhuoyu Long

**Methodology:** Hui Cao, Zhaoxu Huang, Hua Yuan, Xuehui Hu, Zhanli Fu, Meixia Zhang, Zhuoyu Long

**Writing – original draft:** Hui Cao, Xuehui Hu, Zhuoyu Long

**Writing – review & editing:** Hui Cao, Xuehui Hu, Zhuoyu Long

**Validation:** Xuehui Hu, Zhanli Fu, Xiaohuan Song

**Funding acquisition:** Xiaohuan Song, Zhuoyu Long

**Supervision:** Meixia Zhang, Zhuoyu Long
